# The 3T3-L1 adipocyte glycogen proteome

**DOI:** 10.1186/1477-5956-11-11

**Published:** 2013-03-22

**Authors:** David Stapleton, Chad Nelson, Krishna Parsawar, Marcelo Flores-Opazo, Donald McClain, Glendon Parker

**Affiliations:** 1 Department of Physiology, The University of Melbourne, Parkville, VIC, Australia; 2 Mass Spectrometry and Proteomics Core Facility, University of Utah, Rm 5C124 SOM, 30 N 1900 E, Salt Lake City, Utah, 84132, USA; 3 University of Utah School of Medicine, Rm 4C464B SOM, 30 N 1900 E, Salt Lake City, Utah 84132, USA; 4 Department of Biology, Utah Valley University, 800 West University Parkway, Orem, UT 801-863-6907, USA

**Keywords:** Glycogen, Glycogen-associated proteins, 3T3-L1 adipocytes, Proteomics, 14-3-3 proteins, Protein phosphatase 1 regulatory subunit 3D

## Abstract

**Background:**

Glycogen is a branched polysaccharide of glucose residues, consisting of α-1-4 glycosidic linkages with α-1-6 branches that together form multi-layered particles ranging in size from 30 nm to 300 nm. Glycogen spatial conformation and intracellular organization are highly regulated processes. Glycogen particles interact with their metabolizing enzymes and are associated with a variety of proteins that intervene in its biology, controlling its structure, particle size and sub-cellular distribution. The function of glycogen in adipose tissue is not well understood but appears to have a pivotal role as a regulatory mechanism informing the cells on substrate availability for triacylglycerol synthesis. To provide new molecular insights into the role of adipocyte glycogen we analyzed the glycogen-associated proteome from differentiated 3T3-L1-adipocytes.

**Results:**

Glycogen particles from 3T3-L1-adipocytes were purified using a series of centrifugation steps followed by specific elution of glycogen bound proteins using α-1,4 glucose oligosaccharides, or maltodextrins, and tandem mass spectrometry. We identified regulatory proteins, 14-3-3 proteins, RACK1 and protein phosphatase 1 glycogen targeting subunit 3D. Evidence was also obtained for a regulated subcellular distribution of the glycogen particle: metabolic and mitochondrial proteins were abundant. Unlike the recently analyzed hepatic glycogen proteome, no endoplasmic proteins were detected, along with the recently described starch-binding domain protein 1. Other regulatory proteins which have previously been described as glycogen-associated proteins were not detected, including laforin, the AMPK beta-subunit and protein targeting to glycogen (PTG).

**Conclusions:**

These data provide new molecular insights into the regulation of glycogen-bound proteins that are associated with the maintenance, organization and localization of the adipocyte glycogen particle.

## Background

Glycogen is a large intracellular particle consisting of a glycogenin core-protein and a branched glucose polysaccharide covalently attached to a C-1-O-tyrosyl linkage at tyrosine 194 [1]. The α1,4 glucosidic polymer consists primarily of oligosaccharides of 10 to 14 residues in length linked to other oligosaccharides via α1,6 branch points. The resulting three-dimensional structure contains up to 12 oligosaccharide ‘layers’ with 50 to 60 thousand glucose residues and a final mass of about 10^7^ Da [2]. Glycogen comprises smaller glycogen β particles 20–50 nm in diameter, depending on the tissue, and can also form much larger rosettes denoted α particles (100–300 nm in diameter) [3,4]. The function of glycogen is to efficiently store and release glucose monosaccharides in a manner that is rapidly accessible to the metabolic and synthetic requirements of the cell [2,5].

Glucose flux into and out of the polysaccharide is controlled by a combination of substrate availability and regulation of catalytic activities, particularly the rate-limiting enzymes glycogen synthase and glycogen phosphorylase [2]. Control of glycogen branching structure, necessary for prolonged unidirectional glucose flux, is controlled by both glycogen branching enzyme and glycogen debranching enzyme. In addition to catalytic activity, each of these enzymes also has carbohydrate binding activity, containing glycogen or starch-binding domains known as carbohydrate binding modules (CBM); http://www.cazy.org). The glycogen polysaccharide, therefore, physically associates with all of the primary enzymes controlling initiation and dynamic turnover of the particle [6-9]. In addition, regulatory proteins such as phosphorylase kinase, laforin, and protein phosphatase 1 glycogen-targeting subunits are also documented to specifically interact with the glycogen polysaccharide [10-12]. The resulting carbohydrate / protein complex therefore has all of the ingredients to control glucose flux into or out of the molecule, matching the physiological context of the cell or organism [10,13,14].

The release of glucose from intracellular sources complements the elaborate mechanisms of glucose transport into the cell. Together both glucose sources control the concentration of glucose-6-phosphate (G6P), which influences flux into major metabolic and synthetic pathways. Because of its central role in cellular glycogen metabolism is subject to sophisticated, redundant and coordinated kinase signaling pathways. The effects of this regulation are modulated by allosteric factors, particularly the upstream substrate G6P, but also AMP and ATP. Additional regulatory mechanisms, such as O-linked N-acetylglucosamine (O-GlcNAc), 14-3-3 proteins, ubiquitination, and glycogen phosphorylation have also been identified [9,15-17].

Glycogen metabolism is spatially regulated [18,19]. Electron microscopic studies have demonstrated association of glycogen particles with subcellular structures, such as the sarcoplasmic reticulum, the smooth endoplasmic reticulum, other endoplasmic membranes, mitochondria, cytoskeletal elements, and the plasma membrane [18]. The association of glycogen with these structures is dynamic [7]. Recent data have demonstrated a finer sub-organelle control of glycogen localization [19]. There is also physiological evidence of spatial regulation. Glucose channeling into glycogen from gluconeogenesis is more efficient than glucose transported from extracellular sources, indicating that pools of G6P from intracellular sources are more likely to spatially overlap with the glycogen particle [24-26]. The high number of kinase pathways, additional modes of regulation of glycogen metabolic enzymes, and evidence of fine spatial organization of the glycogen particle, together suggest the potential for identification of additional regulatory proteins of glycogen metabolism. We hypothesize that a systematic proteomic analysis of glycogen-associated proteins will identify these proteins.

Adipose tissue is a primary site for energy provision via the hydrolysis of stored triglyceride to release free fatty acid (FFA) for ATP production and glycerol for hepatic gluconeogenesis in addition to being the storage site for dietary lipid. Postprandial glucose is stored as glycogen in the liver and used as an energy source in peripheral tissues but in adipocytes is for *de novo* lipogenesis and long-term storage as triglyceride. However, adipocytes also store glucose as glycogen, albeit at substantially lower rates than in skeletal muscle and liver [27]. The main role for adipose tissue glycogen is believed to yield precursors for glycerol formation especially following a period of fasting where glycogen synthesis increases prior to lipid deposition [28]. This spike in glycogen synthesis is believed to provide substrate for the expansion of adipose mass [29]. However, the involvement of glycogen stores during either obesity or insulin resistance has not been determined. Given the importance of adipocyte glycogen in lipid synthesis we hypothesized that the adipocyte glycogen proteome would include specialized regulatory proteins not found in the liver glycogen proteome whose primary function is to store and breakdown glycogen for the maintenance of blood glucose levels.

In this study we purified glycogen particles from differentiated 3T3-L1-adipocytes. Unlike adipose tissue that has low levels of glycogen, 3T3-L1-adipocytes have high levels of glycogen, are metabolically responsive, and are amenable to *in vitro* manipulation [15]. We purified glycogen particles using a series of centrifugation steps followed by specific elution of glycogen bound proteins using α1,4 glucose oligosaccharides, or maltodextrins. Several regulatory proteins were identified, including 14-3-3 proteins, RACK1 and protein phosphatase 1 glycogen targeting subunit 3D. Evidence was also obtained for a regulated subcellular distribution of the glycogen particle: metabolic and mitochondrial proteins were abundant. Unlike the recently analyzed hepatic glycogen proteome, no endoplasmic proteins were detected [19]. Other regulatory proteins, which have previously been described as glycogen-associated proteins, were not detected, including laforin, AMP-activated protein kinase (AMPK) and protein targeting to glycogen (PTG) [30-32]. We also note that a population of glycogen synthase binds with high affinity to the glycogen particle, even in the presence of high concentrations of malto-oligosaccharides. Together these data provide a proteomic context for analysis of potential and established regulatory mechanisms and further elucidate the role of adipocyte glycogen metabolism in cellular energy homeostasis.

## Results and discussion

### Isolation and processing of glycogen particles

**Figure 1:**
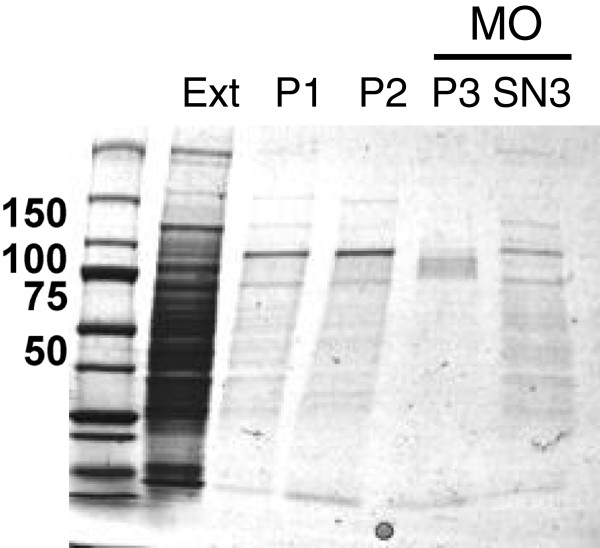
**Purification of the adipocyte glycogen protoeme. **Differentiated 3T3-L1 adipocytes were treated for 24 h with 2.5 mM glucose and 10 mM glucosamine, collected, freeze / thawed, sonicated and the 20 000 g supernatant (Ext) centrifuged at 400 000 g for 30 min. The glycogen pellet (P1) was resuspended and the process was repeated. The second glycogen pellet (P2) was resuspended in 50 mg malto-oligosaccharide (MO) / ml. The MO supernatant (SN3), or soluble fraction, was the reference preparation of glycogen-associated proteins used in this study. The MO pellet (P3), or insoluble fraction, was also trypsinized to provide a control comparative dataset to allow analysis of the stringency of the purification. The proteins illustrated above are a representative purification. Total amounts applied to the coomassie-G250 stained SDS-PAGE above, are 0.05% (Ext) or 2% (P1, P2, P3 and SN3) of the total sample.

Glycogen-associated proteins from mouse 3T3-L1-adipocytes were isolated after repeated centrifugation steps, which were sufficient to obtain a stable protein population associated with the glycogen pellet (Figure 1). The preparation was then treated with malto-oligosaccharides, which are identical to the α1,4 glucose oligosaccharide component of glycogen molecules. This treatment disrupted specific lectin-like interactions of glycogen-associated proteins with the glycogen polysaccharide resulting in solubilization (SN3, Figure 1). The dominant bands remaining in the pellet, which were not solubilized by the malto-oligosaccharide treatment, were determined to be muscle glycogen synthase by immunoblotting (data not shown).

**Figure 2:**
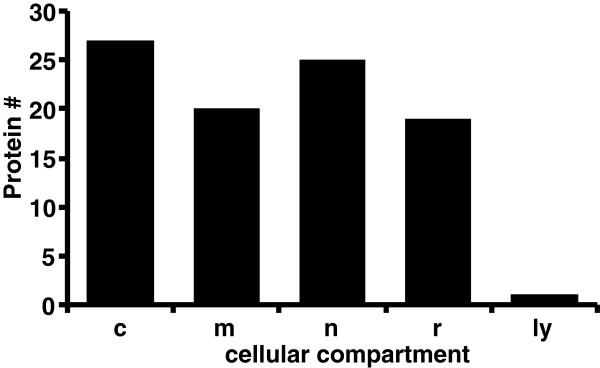
**Cellular distribution of glycogen-associated proteins. **Proteins identified as glycogen-associated proteins, were submitted to the UNIPROT database and the number of identified proteins in each cellular and functional compartments obtained. Abbreviations include: glycogen metabolic (g), cytoplasmic (c), mitochondrial (m), nuclear (n), spliceosomal (s), ribosomal (r), and lysosomal (ly) compartments.

A total of 6 preparations of glycogen-associated proteins, representing biological replicates, were specifically solubilized with malto-oligosaccharides, trypsinized and analyzed by reversed-phase liquid chromatography / mass spectrometry / mass spectrometry (LC/MS/MS). All resulting datasets (n = 6) containing collision-induced dissociation (CID) spectra were concatenated and analyzed using the MASCOT software algorithm (http://www.matrixscience.com), the PROWL search engine (http://prowl.rockefeller.edu) and UNIPROT database (http://www.uniprot.org). Identified gene products that contained at least 2 unique peptide sequences with expectation values less than 0.05 are listed functionally in order of likelihood of identification (Table 1), with a complete listing in the Supplemental section (Additional file 1: Table S1). This dataset was analyzed two ways: by function (Table 1) and subcellular distribution (Figure 2). The individual (n = 6) datasets were compared to determine the number of instances a particular protein was repeatedly identified in the glycogen-associated population (Table 2). To further evaluate and identify potentially contaminating proteins, datasets from 3 pellets, after specific elution using α1,4 glucose oligosaccharides, were concatenated and analyzed (Tables 3 and 4) [33,34].

**Table 1:** The adipocyte glycogen proteome

**Name**	**ID**	**Score**	**Total**	**#**	**%**	**E-value**	**EmPAI:**
**Glycogen**							
Glycogen phosphorylase, brain form	PYGB_MOUSE	20722	599	55	56.5	3.70E-10	17.06
Glycogen synthase, muscle	Q8VEB0_MOUSE	7954	283	39	57.2	8.50E-09	7.67
Glycogen branching enzyme	GLGB_MOUSE	4302	141	28	37.0	2.20E-08	4.30
Glycogen phosphorylase, muscle form	PYGM_MOUSE	3687	138	16(1)	18.3	5.30E-02	1.11
Glycogen debranching enzyme*	GDE_RABIT	3387	115	20	14.9	9.10E-09	0.67
Glycogenin-1	GLYG_MOUSE	1394	49	9	22.3	1.40E-06	2.17
Protein phosphatase 1, regulatory subunit 3D (or R6)	A2AJW4_MOUSE	701	17	4	15.1	4.80E-08	0.55
Lysosomal alpha-glucosidase	LYAG_MOUSE	102	2	2	2.9	5.30E-06	0.07
Serine/threonine-protein phosphatase PP1-alpha catalytic subunit	PP1A_MOUSE	76	3	2	8.8	6.50E-04	0.24
**Metabolism**							
Glyceraldehyde-3-phosphate dehydrogenase	G3P_MOUSE	1311	42	10	42.6	1.20E-07	2.01
Pyruvate carboxylase	PYC_MOUSE	1182	31	11	12.3	1.60E-08	0.40
Malate dehydrogenase	MDHM_MOUSE	827	16	4	20.4	2.90E-09	0.49
ATP synthase subunit beta	ATPB_MOUSE	619	16	9	24.5	3.90E-08	0.94
Aldehyde dehydrogenase	ALDH2_MOUSE	513	15	3	7.5	4.00E-07	0.22
ATP synthase subunit alpha	ATPA_MOUSE	441	17	7	17.5	2.30E-05	0.62
L-lactate dehydrogenase A chain	LDHA_MOUSE	438	7	3	14.5	4.20E-10	0.34
Alpha-enolase	ENOA_MOUSE	424	10	4	14.1	2.10E-07	0.36
Aconitate hydratase	ACON_MOUSE	368	7	3	6.7	1.70E-09	0.14
Acetyl-CoA acetyltransferase	THIL_MOUSE	343	12	2	14.1	1.00E-09	0.17
(14 other proteins)							
**RNA**							
Cleavage and polyadenylation specificity factor subunit 6	CPSF6_MOUSE	928	16	6	15.8	9.80E-09	0.41
Putative RNA-binding protein Luc7-like 2	LC7L2_MOUSE	633	21	6	18.8	5.10E-07	0.61
Cisplatin resistance-associated overexpressed protein	CROP_MOUSE	529	12	3	17.4	3.40E-08	0.48
Splicing factor, arginine/serine-rich 3	SFRS3_MOUSE	492	16	4	36.3	1.20E-04	2.38
Elongation factor 1-alpha 2	EF1A2_MOUSE	461	15	5	11.5	6.90E-07	0.43
Splicing factor U2AF 65 kDa subunit	U2AF2_MOUSE	460	20	5	19.6	3.20E-05	0.40
Putative RNA-binding protein Luc7-like 1	LUC7L_MOUSE	384	15	3(1)	10.2	3.70E-06	0.32
ATP-dependent RNA helicase DDX3X	DDX3X_MOUSE	361	8	4	8.9	1.10E-07	0.28
Cleavage and polyadenylation specificity factor subunit 5	CPSF5_MOUSE	361	16	6	36.1	6.80E-08	1.57
Pre-mRNA-splicing factor 38B	PR38B_MOUSE	299	9	4	6.8	4.70E-07	0.25
(7 other proteins)							
**Regulatory proteins**							
14-3-3 protein gamma	1433G_MOUSE	795	32	9	33.7	3.90E-07	2.55
14-3-3 protein beta/alpha	1433B_MOUSE	554	27	10(4)	40.8	1.30E-05	2.56
14-3-3 protein zeta/delta	1433Z_MOUSE	488	23	6	26.5	7.90E-04	1.16
14-3-3 protein theta	1433T_MOUSE	265	15	4(1)	14.8	5.40E-05	0.70
14-3-3 protein epsilon	1433E_MOUSE	244	14	3(1)	12.9	2.00E-03	0.49
Receptor of activated protein kinase C 1	GBLP_MOUSE	212	7	2	6.3	6.50E-06	0.23
**Ribosomes**							
40S ribosomal protein SA	RSSA_MOUSE	377	8	2	9.5	4.00E-06	0.24
40S ribosomal protein S16	RS16_MOUSE	278	10	4	24.0	1.30E-05	1.34
40S ribosomal protein S18	RS18_MOUSE	265	10	5	25.7	2.20E-05	1.68
40S ribosomal protein S7	RS7_MOUSE	224	9	2	10.3	9.80E-06	0.38
Nucleolin	NUCL_MOUSE	217	5	3	6.1	7.30E-07	0.15
40S ribosomal protein S2	RS2_MOUSE	214	7	3	11.9	2.60E-05	0.41
40S ribosomal protein S4, X isoform	RS4X_MOUSE	186	5	2	9.9	1.10E-05	0.27
40S ribosomal protein S24	RS24_MOUSE	184	6	2	20.3	5.20E-05	0.57
40S ribosomal protein S8	RS8_MOUSE	182	5	2	12.5	9.80E-05	0.34
40S ribosomal protein S13	RS13_MOUSE	150	5	3	24.5	1.30E-04	0.84
(12 other proteins)							
**Miscellaneous**							
Major vault protein	MVP_MOUSE	1271	41	13	23.9	1.30E-07	0.69
Histone H2B type 1-B †	H2B1B_MOUSE	881	17	3	27.2	2.00E-09	1.73
Stress-70 protein	GRP75_MOUSE	789	28	7	13.8	3.00E-06	0.41
Histone H2A type 1-F†	H2A1F_MOUSE	556	19	3	43.8	6.80E-07	1.67
60 kDa heat shock protein	CH60_MOUSE	343	12	7	14.1	8.00E-07	0.63
Histone H1.2 †	H12_MOUSE	291	10	4	15.1	3.90E-05	0.91
10 kDa heat shock protein	CH10_MOUSE	238	4	2	19.6	9.50E-09	0.86
Histone H4 †	H4_MOUSE	222	8	5	47.6	9.60E-06	3.51
Actin, alpha skeletal muscle	ACTS_MOUSE	101	3	2	16.5	3.10E-04	0.19
Histone H2A.x †	H2AX_MOUSE	95	6	3(1)	47.3	1.80E-02	1.11
Tubulin beta-2B chain	TBB2B_MOUSE	93	3	3	11.0	1.20E-03	0.24

Proteins isolated from glycogen particles of mouse 3T3-L1 adipocytes were solubilized by malto-oligosaccharide treatment and trypsinized. Peptides were desalted, purified, and analyzed by mass spectrometry (LC/MS/MS) and bioinformatic searches of the “mammalia” MSDB database. Mouse proteins, identified by name and UNIPROT identifier (ID), that meet a significance level of less than 0.05 and with at least two different unique peptide sequences with expectation scores less than 0.05, are listed functionally in order of likelihood by Mowse score (score). Other parameters include: the total number of peptides with expectation scores less than 0.05 (total), number of different unique peptide sequences identified corresponding to the specific gene product (#), percent coverage of the protein (%), and the lowest recorded expectation value (E-value) for a unique non-redundant peptide from the identified protein. In the case of proteins with homology to other glycogen-associated proteins, the number of unique, non-redundant peptides is indicated in parentheses. Exponentially modified Protein Abundance Index (emPAI) values are also included [35]. *The UniProt listing for GDE-MOUSE is not complete therefore the parameters presented correspond to the closet match in the database, GDE_RABIT. †Insufficient data was obtained to conclusively match the identified peptides with a unique gene product. A complete listing of proteins and parameters is located in the Supplemental section (Additional file 1: Table S1).

**Table 2:** Repeated occurrence of proteins in the glycogen-associated proteome

**Name**	**Gene**	**#**
Glycogen phosphorylase, brain form	PYGB_MOUSE	6
Glycogen branching enzyme	GLGB_MOUSE	6
Glycogen synthase, muscle	Q8VEB0_MOUSE	6
Glycogen debranching enzyme	GDE_RABIT	5
Glycogenin-1	GLYG_MOUSE	4
Glyceraldehyde-3-phosphate dehydrogenase	G3P_MOUSE	4
Histone H2B F	H2B1_MOUSE	4
14-3-3 protein beta/alpha	1433B_MOUSE	3
Fatty acid synthase	FAS_MOUSE	3
Aconitate hydratase, mitochondrial	ACON_MOUSE	3
Malate dehydrogenase	MDHM_MOUSE	3
Fatty acid-binding protein, adipocyte	FABPA_MOUSE	3
Dihydrolipoamide acyltransferase (E2)	Q8BMF4_MOUSE	3
60S acidic ribosomal protein P1	RLA1_MOUSE	3
Actin beta	ACTB_MOUSE	3
GRP 75	GR75_RAT	3
60 kDa heat shock protein	CH60_MOUSE	3
Major Vault Protein	MVP_MOUSE	3
Isocitrate dehydrogenase subunit alpha, mitochondrial	IDH3A_MOUSE	3
10 kDa chaperonin C	Q9JI95_MOUSE	3
Keratin Kb40	Q6IFT3_MOUSE	3
Keratin, type II cytoskeletal 1b	K2C1B_MOUSE	3
Keratin, type II cytoskeletal 1	K22E_MOUSE	3
Ribonuclease UK114	UK14_MOUSE	3
Trypsin - pig	TRYP_PIG	3

Six preparations of mouse 3T3-L1 adipocyte glycogen-associated, malto-oligosaccharide solubilized proteins were trypsinized, desalted, purified, and analyzed by mass spectrometry (LC/MS/MS). The resulting six datasets of fragmentation spectra were matched with peptide sequences using the MASCOT search algorithm and the “Rodentia” MSDB database. The number of times that a protein, identified by name and UNIPROT identifier (ID), appeared in the six datasets is listed (#). Identified proteins met a significance level of less than 0.05 and had at least two different unique peptide sequences with expectation scores less than 0.05.

**Table 3:** Proteins present in glycogen pellet following specific elution with malto-oligosaccharides

**Name**	**ID**	**Score**	**Total**	**#**	**%**	**E-value**	**EmPAI**
Glycogen synthase 1, muscle	Q8VEB0_MOUSE	7920	254	29	41.7	8.20E-10	7.67
Glycogen phosphorylase, brain form	PYGB_MOUSE	4197	122	23	33.8	2.70E-09	1.76
Glycogenin-1	GLYG_MOUSE	1360	39	7	21.7	1.40E-08	1.17
Splicing factor, arginine/serine-rich 3	SFRS3_MOUSE	1197	43	4	36.3	9.00E-06	3.31
Cisplatin resistance-associated overexpressed protein	CROP_MOUSE	985	19	5	28.8	1.10E-08	0.93
Cleavage and polyadenylation specificity factor subunit 6	CPSF6_MOUSE	900	13	3	8.8	1.20E-10	0.26
Glyceraldehyde-3-phosphate dehydrogenase	G3P_MOUSE	753	19	5	23.4	2.90E-07	0.65
Splicing factor U2AF 65 kDa subunit	U2AF2_MOUSE	727	34	5	19.6	1.50E-06	0.60
Major vault protein	MVP_MOUSE	726	25	11	16.6	2.50E-05	0.52
Putative RNA-binding protein Luc7-like 2	LC7L2_MOUSE	623	23	6	17	1.60E-06	0.61
ATP synthase subunit beta	ATPB_MOUSE	620	16	6	17.7	1.90E-07	0.55
ATP-dependent RNA helicase DDX3X	DDX3X_MOUSE	552	13	5	10.7	1.00E-07	0.35
ATP synthase subunit alpha	ATPA_MOUSE	530	14	7	18.8	1.90E-06	0.53
Cleavage and polyadenylation specificity factor subunit 5	CPSF5_MOUSE	412	14	5	27.8	3.90E-08	1.58
Splicing factor, arginine/serine-rich 7	SFRS7_MOUSE	409	11	3(2)	22.3	1.30E-06	1.19
Histone H2A type 1-F †	H2A1F_MOUSE	403	13	2	21.5	1.60E-07	1.09
Elongation factor 1-alpha 1	EF1A1_MOUSE	368	12	3	6.3	1.70E-07	0.24
Putative RNA-binding protein Luc7-like 1	LUC7L_MOUSE	355	16	3(1)	10.2	1.40E-02	0.32
Pre-mRNA-splicing factor 38B	PR38B_MOUSE	348	14	3	6.8	3.80E-07	0.18
60 kDa heat shock protein	CH60_MOUSE	323	8	3	8.3	3.90E-07	0.28
Histone H2B type 1-F/J/L †	H2B1F_MOUSE	260	4	2	19.2	1.70E-09	0.66
Splicing factor U2AF 35 kDa subunit	U2AF1_MOUSE	253	7	2	13	4.30E-05	0.29
Heterogeneous nuclear ribonucleoprotein H2	HNRH2_MOUSE	247	7	3(1)	9.6	6.40E-05	0.24
Stress-70 protein	GRP75_MOUSE	242	6	3	6.3	2.80E-06	0.16
Heterogeneous nuclear ribonucleoprotein M	HNRPM_MOUSE	234	7	35	8	1.20E-05	0.21
Trifunctional enzyme subunit alpha	ECHA_MOUSE	226	7	4	8.7	2.50E-05	0.19
Histone H1t †	H1T_MOUSE	213	6	2	10.5	1.90E-06	0.38
Vimentin	VIME_MOUSE	163	3	2	5.2	1.10E-07	0.14
Probable ATP-dependent RNA helicase DDX5	DDX5_MOUSE	148	4	3(2)	4.3	6.70E-05	0.15
Probable ATP-dependent RNA helicase DDX17	DDX17_MOUSE	145	4	2(1)	3.5	9.90E-05	0.10
40S ribosomal protein S7	RS7_MOUSE	126	4	2	10.3	9.90E-06	0.38
60S ribosomal protein L23a	RL23A_MOUSE	115	4	3	19.9	2.40E-04	0.81
Tubulin beta-2B chain †	TBB2B_MOUSE	109	5	2	5.4	1.60E-02	0.24
Pyruvate carboxylase	PYC_MOUSE	104	4	3	3.8	2.40E-04	0.09
Glycogen debranching enzyme *	GDE_HUMAN	104	3	2	2	3.50E-04	0.04
Glycogen debranching enzyme *	Q8CE68_MOUSE	103	3	2(1)	1.9	2.90E-04	0.04
Ubiquitin	UBIQ_MOUSE	87	3	3	44.7	1.60E-04	2.23
60S ribosomal protein L11	RL11_HUMAN	81	2	2	13.8	5.00E-05	0.45
ATP synthase subunit O	ATPO_MOUSE	68	2	2	14.1	3.40E-03	0.35
Histone H4 †	H4_MOUSE	64	2	2	19.4	3.40E-03	0.83

Proteins that were still present in the glycogen pellet after specific treatment with malto-oligosaccharides are listed (name and UNIPROT identifier (ID)) in order of likelihood of identification by Mowse score (score). This population represents proteins whose physical interaction with the glycogen molecule is not disrupted by malto-oligosaccharide treatment either because of non-specific interaction with the particle or because the specific interaction is not adequately competed with by the elution conditions used, 50 mg malto-oligosaccharide / ml. Other parameters included in the table are: the total number of unique non-redundant peptide sequences identified (#), per cent coverage of identified proteins (%), and minimum expectation value for a unique non-redundant peptide from within the identified gene product (E-value). Exponentially modified Protein Abundance Index (emPAI) values are also included [35]. Peptides with expectation scores above 0.05 were included in the analysis. All proteins listed have a minimum of two unique non-redundant peptides identified. †Insufficient data was obtained to conclusively match the identified peptides with a unique gene product.

**Table 4:** comparison of malto-oligosaccharide soluble and insoluble populations

**Name**	**ID**	**Sol**	**Insol**	**Sol/insol**	**Compartment**
Glycogen-branching enzyme	GLGB_MOUSE	0.25	0.03	**7.39**	g
Glycogen debranching enzyme *	GDE_HUMAN	0.04	0.02	**1.73**	g
Protein phosphatase 1 regulatory subunit 3D	A2AJW4_MOUSE	0.03	0.03	**1.13**	g
**Glycogen phosphorylase, brain form**	PYGB_MOUSE	1.00	1.00	**1.00**	g
Pyruvate carboxylase	PYC_MOUSE	0.02	0.05	**0.46**	m
Histone H4 †	H4_MOUSE	0.21	0.47	**0.44**	n
Histone H2B type 1-F/J/L †	H2B1F_MOUSE	0.15	0.38	**0.40**	n
Glyceraldehyde-3-phosphate dehydrogenase	G3P_MOUSE	0.12	0.37	**0.32**	c
Stress-70 protein	GRP75_MOUSE	0.02	0.09	**0.26**	m
Lysosomal alpha-glucosidase	LYAG_MOUSE	0.00	0.02	**0.24**	g
60 kDa heat shock protein	CH60_MOUSE	0.04	0.16	**0.23**	m
Probable ATP-dependent RNA helicase DDX17	DDX17_MOUSE	0.01	0.06	**0.23**	s
Glycogenin-1	GLYG_MOUSE	0.13	0.66	**0.19**	c
Probable ATP-dependent RNA helicase DDX5	DDX5_MOUSE	0.02	0.09	**0.19**	s
ATP synthase subunit beta	ATPB_MOUSE	0.06	0.31	**0.18**	m
Cleavage and polyadenylation specificity factor subunit 6	CPSF6_MOUSE	0.02	0.15	**0.16**	s
Histone H2A type 1-F †	H2A1F_MOUSE	0.10	0.62	**0.16**	n
Heterogeneous nuclear ribonucleoprotein H2	HNRH2_MOUSE	0.02	0.14	**0.15**	s
Pre-mRNA-splicing factor 38B	PR38B_MOUSE	0.01	0.10	**0.14**	s
Major vault protein	MVP_MOUSE	0.04	0.30	**0.14**	c
Heterogeneous nuclear ribonucleoprotein M	HNRPM_MOUSE	0.02	0.12	**0.13**	s
ATP synthase subunit alpha	ATPA_MOUSE	0.04	0.30	**0.12**	m
Glycogen synthase 1, muscle	Q8VEB0_MOUSE	0.45	4.36	**0.10**	g
Tubulin beta-2B chain †	TBB2B_MOUSE	0.01	0.14	**0.10**	c
ATP synthase subunit O	ATPO_MOUSE	0.02	0.20	**0.10**	m
Putative RNA-binding protein Luc7-like 2	LC7L2_MOUSE	0.04	0.35	**0.10**	s
Putative RNA-binding protein Luc7-like 1	LUC7L_MOUSE	0.02	0.18	**0.10**	s
Cleavage and polyadenylation specificity factor subunit 5	CPSF5_MOUSE	0.09	0.90	**0.10**	s
Trifunctional enzyme subunit alpha	ECHA_MOUSE	0.01	0.11	**0.09**	m
ATP-dependent RNA helicase DDX3X	DDX3X_MOUSE	0.02	0.20	**0.08**	s
Splicing factor, arginine/serine-rich 3	SFRS3_MOUSE	0.14	1.88	**0.07**	s
Splicing factor U2AF 65 kDa subunit	U2AF2_MOUSE	0.02	0.34	**0.07**	s
Cisplatin resistance-associated overexpressed protein	CROP_MOUSE	0.03	0.53	**0.05**	s

The final purification of glycogen-associated proteins involved elution with malto-oligosaccharides, which competes with the glycogen molecule for specific interactions with the glycogen-associated proteins. To provide a measure of specific interaction with the glycogen molecule, proteins common to both the malto-oligosaccharide soluble and insoluble datasets were compared. Exponentially modified Protein Abundance Index (emPAI) values for each protein in the malto-oligosaccharide soluble (sol) and insoluble (insol) datasets were divided by the emPAI value for glycogen phosphorylase, a reference protein known to specifically interact with the glycogen molecule [10,35]. These relative measures of binding to the glycogen particle were then directly compared: sol / insol = ((emPAI of protein in soluble dataset / emPAI of soluble glycogen phosphorylase) / (emPAI of protein in insoluble dataset / emPAI of insoluble glycogen phosphorylase)). This calculation provides a measure of relative binding to the glycogen molecule and allows comparison between differently sized datasets. Proteins are listed in order of relative solubility with malto-oligosaccharides (sol/insol). The reference protein, glycogen phosphorylase, is indicated in bold. Values greater than one represent proteins that are relatively more soluble than glycogen phosphorylase. The subcellular localization of proteins common to both soluble and insoluble datasets is also listed (compartment).

### Identification of glycogen-associated proteins

The proteomic methodology was validated by the consistent and frequent identification of proteins previously known to maintain and physically associate with the glycogen particle [10,13,14,36]. For instance, all glycogen metabolic enzymes were present: glycogen phosphorylase, brain isoform (GP; Mowse score = 20722), glycogen synthase, muscle isoform (GS; score = 7954), glycogen branching enzyme (GBE; score = 4302), glycogen debranching enzyme (GDE; score = 3387), and glycogenin-1 (score = 1394) [10,13,14,36]. The muscle isoform of glycogen phosphorylase (score = 3687) was also present, indicating that brain and muscle GP are involved in the regulation of cytoplasmic glycogenolysis in these cells. Known regulatory proteins were also identified, such as protein phosphatase 1 catalytic subunit (score = 76) and the glycogen targeting subunit 3D (PPP1R6 also known as R6; score = 701). Other known glycogen-associated proteins, such as laforin, AMPK, and PTG were not detected [10]. The absence of AMPK could be explained by the predominant expression of the β_1_-subunit in adipose tissue, instead of the AMPK β_2_-subunit that has a 10- to 30-fold increased affinity for linear or branched oligosaccharides [31,40,41]. A single peptide from starch-binding domain protein 1 (STBD1) and phosphorylase kinase was detected, insufficient to meet the criteria as a glycogen-associated protein in this analysis (data not shown). Glycogenin-1 was relatively abundant indicating that a population of this protein was accessible during trypsin digestion [42]. Glycogenin-1 is necessary for initiating glycogen synthesis and therefore is located at the center of each glycogen particle [2]. Association of glycogenin-1 with the outer surface, as shown in this study and in another study on the hepatic glycogen proteome, was therefore unexpected [19]. Lysosomal alpha-glucosidase was also identified in the data set (score = 102). This enzyme is necessary for the lysosomal degradation of the glycogen particle and is the gene product that accounts for Glycogen Storage Disease II (or Pompe’s disease) [43,44].

A predominant glycogen targeting regulatory subunit of phosphatase I was identified in the study, PPP1R6 (or 3D) (Table 1), found previously associated with glycogen from skeletal muscle [45]. The presence of PPP1R6 in adipocytes is a new finding and may suggest an important role for this PP1 targeting subunit in the adipocyte. Unexpectedly, the analyses failed to detect any peptides from the gene product Protein Targeted to Glycogen (PTG; or regulatory subunit 3C, or R5) [46,47]. RNAi-mediated reduction of PTG in 3T3-L1 adipocytes decreased glycogen accumulation, indicating a central role for PTG in glycogen metabolism [48]. This is supported by studies in knockout mice, which exhibit a phenotype of reduced adipose glycogen levels, although there are different effects on glycogen metabolism and insulin resistance [49] (Anna DePauli-Roach, personal communication). As with other unexpectedly absent gene products, such as laforin or AMPK, the possibility exists that control over glycogen metabolism can be exerted by proteins of low abundance, below the level of detection, or by proteins that have low affinity for the glycogen particle. Glycogen synthase was relatively resistant to solubilization from the glycogen particle, with α1,4 glucose oligosaccharide treatment, implying either that a structural form of glycogen synthase has higher affinity for the particle or, alternatively, glycogen synthase may bind to a motif that is dissimilar to the α1,4 glucose oligosaccharide.

Many proteins with no documented role in glycogen metabolism were also found to be associated with glycogen (Table 1). The largest functional group was metabolic enzymes not directly involved in glycogen metabolism, with either mitochondrial or cytoplasmic origins, the latter being primarily glycolytic enzymes (24 total, Table 1). As anticipated, given the cell source, some enzymes involved in lipid metabolism were also detected, such as acetyl-CoA acetyltransferase (THIL_MOUSE, Table 1).

As predicted, regulatory proteins were also identified. For example, receptor of activated protein kinase C 1 (RACK1) is a scaffolding protein that binds to protein kinases and membrane-bound receptors in a regulated fashion. It targets protein kinase C to ribosomes and hypoxia-inducible factor 1 to the proteosome [50,51]. Five isoforms of 14-3-3 proteins were unambiguously identified in this study. 14-3-3 proteins have a wide range of regulated interaction with phosphoproteins, contributing to many cellular processes including carbohydrate metabolism [52,53]. The most abundant isoform, 14-3-3 gamma, has been linked to the development of obesity in humans [54]. The beta-isoform of 14-3-3 is also named protein kinase C inhibitor protein 1. This is the first description of 14-3-3 proteins associating with the glycogen particle. A recent analysis of the hepatic glycogen proteome did not identify these regulatory proteins, indicating a level of specificity for these proteins in the adipocyte glycogen complex [19].

### Comparison of malto-dextrin soluble and insoluble glycogen-associated protein populations

The purification of glycogen particles depends on a series of ultracentrifuge steps, leaving open the possibility that the presence of ribosomes, spliceosomes, and vault proteins could be due to co-purification through co-precipitation [55,56]. This is confirmed by the increased relative abundance of these proteins in the glycogen pellet after the malto-oligosaccharide treatment, potentially excluding these proteins as part of the glycogen-associated population (Tables 3 and 4).

The relative solubility, or insolubility, of proteins in the presence of malto-oligosaccharides was estimated by determining the relative abundance of each protein relative to glycogen phosphorylase [35]. The quotient of these values provides a measure of specificity for binding to the glycogen macromolecule. In Table 4, all proteins that were relatively more soluble than glycogen phosphorylase, with a quotient greater than 1.00, were found to be glycogen metabolic proteins. Glycogen synthase and glycogenin were more associated with the glycogen pellet than glycogen phosphorylase. These enzymes are known to specifically interact with the glycogen particle in a targeted manner. In this case, enrichment in the glycogen pellet may have been due to the inability of α1,4 glucose oligosaccharides to interact with and solubilize these proteins. This population of totally α1,4 glucose oligosaccharide-solubilized proteins includes regulatory proteins, the 14-3-3-isoforms, and RACK1. This indicates that these proteins are candidates for a population that specifically associates with the glycogen protein / carbohydrate complex and potentially play a role in protein regulation. This is the first description of these proteins as candidate members of the glycogen proteome.

### Sub-cellular distribution of glycogen-associated proteins

When subcellular locations of glycogen-associated proteins are assigned, as annotated in the UNIPROT database (http://uniprot.org), a distinctive distribution was observed (Figure 2). Glycogen particles have been documented in the cytoplasm, and with mitochondria, ribosomes, and endoplasmic and other membranes [10,13,19,21,57,58]. This corresponds to adipocyte glycogen-associated proteins that derive predominantly from the cytoplasm, mitochondria, or ribosomes. The population is also rich in nuclear proteins, particularly spliceosomal proteins and histones, which we do not consider to be specifically associated with the particle (Table 4). The homogenization process disrupts the intracellular architecture, physically disrupting the glycogen particle along with attached, fragmented cellular structures. These cytoskeletal elements, membranes and complexes with the associated proteins would then co-purify.

There are no endoplasmic reticulum proteins present in the adipocyte glycogen proteome (Figure 2). This is in contrast to the hepatic glycogen-associated proteome, where proteins associated with the endoplasmic reticulum were highly abundant [19]. This was consistent with the demonstration of starch binding domain protein-1 (STBD-1) (or genethonin-1) as a glycogen-associated protein in liver [19]. This protein contains a glycogen-binding domain and an endoplasmic reticulum-targeting transmembrane domain, the predicted primary structure for a protein mediating glycogen association with membranes [34,59]. Only a single peptide of this protein was detected in this analysis, insufficient for inclusion in this dataset. The absence of endoplasmic reticulum-associated proteins in the adipocyte glycogen population indicates that targeting of glycogen to subcellular sites is different in different cell types [19,57,58,60,61]. In addition, STBD1 is now thought to be involved in glycogen autophagy and given that STBD1 is expressed in adipose tissue, the low level of this protein in our proteomic data, only a single peptide, suggests that adipose glycogen may not be degraded by this process2glycogen [2,63,64].

## Conclusion

The 3T3-L1 adipocyte glycogen proteome consists of enzymes essential for its synthesis together with specific regulatory proteins PPP1R6, RACK1 and the family of 14-3-3 protein isoforms, the most abundant of which have been associated with obesity [64,65]. This is the first description of the latter proteins as being potentially associated with glycogen particles. Evidence of associating mitochondrial proteins and a lack of endoplasmic reticulum proteins suggest a different spatial arrangement of adipocyte glycogen particles compared to hepatic glycogen particles. These data provide new molecular insights into the relationship of adipocyte glycogen metabolism with other cellular processes and can be expanded to provide a starting point for analyzing the glycogen proteome in adipose tissue from animal models of Type 2 Diabetes and obesity. This will lead to the identification of novel mechanisms and protein activities that control the organization and deposition of glucose metabolism as well as its integration into cellular biosynthetic and metabolic pathways.

## Methods

### Preparation and trypsinization of 3T3-L1 adipocyte glycogen-associated proteins

3T3-L1 cells (American Type Culture Collection, Manassas, VA) were differentiated into 3T3-L1-adipocytes as described previously [15]. Adipocytes were treated for 24 h in 10% fetal bovine serum, and 10 ml of DMEM containing 10 Units of penicillin, 10 μg of streptomycin, 29.2 μg glutamine, 2.5 mM glucose and 10 mM glucosamine, conditions that maximize glycogen accumulation and simulate energy repletion [15,66]. Cells were scraped and collected in 750 μl of the extraction buffer (50 mM HEPES 7.4, 100 mM NaCl, 50 mM NaF and 50 μM O-(2-Acetamido-2-deoxy-D-glucopyranosylidene) amino N-phenyl carbamate (PUGNAc)) (Toronto Research Chemicals, New York, ON), frozen in liquid N_2_, thawed, sonicated for 15 seconds and centrifuged at 20 000 g for 2 min at 4°C. A stock of 20 mg glycogen type-III / ml was added to the supernatant to give a final concentration of 4 mg / ml, and the sample was centrifuged in polycarbonate centrifuge tubes at 400 000 g for 30 minutes in a Beckman Model TLX 120 ultracentrifuge (Beckman-Coulter, Fullerton, CA). The resulting tubes and pellets were carefully cleaned using sterile cotton swabs and Nanopure water. Pellets were then resuspended in extraction buffer and recentrifuged at 400 000 g for 30 minutes at 4°C. The third pellet preparations were resuspended in extraction buffer and 200 mg α1,4 malto-oligosaccharides / ml was added to give a final concentration of 50 mg / ml (MD-6 Calibrated Standard Maltodextrins, V-labs inc., Covington, LA). The malto-oligosaccharide competes with the glycogen molecule for binding to specifically associated glycogen-immobilized proteins. The sample was thoroughly mixed and recentrifuged at 400 000 g for 30 minutes at 4°C. The supernatant containing malto-oligosaccharide-solubilized glycogen-associated proteins (SN3, Figure 1) was either stored at -20°C or treated immediately by addition of 20 μg TPCK-treated trypsin (Trypsin Gold, Promega, Madison, WI) and incubated at 37°C for 16 h. Tryptic peptides were desalted and purified by C18 ZipTip™ desalting columns (Millipore, Billerica, MA), eluted with 10 μl of 50% methanol, 0.5% acetic acid and 2 μl acetonitrile.

A total of 6 preparations, representing biological replicates, were used in this study. As a comparison, an additional 3 tryptic digests were conducted on the final malto-oligosaccharide-washed glycogen pellet using the conditions described above. All buffers and solutions were passed through a Sep-Pak reversed-phase tC18 solid-phase extraction column (Waters, Milford, MA) to remove contaminating peptides and large organic compounds prior to analysis by mass spectrometry.

### Mass spectrometry

Tryptic preparations of glycogen-associated proteins were analyzed by LC/MS/MS using an ESI Ion-Trap/FTMS hybrid mass spectrometer (LTQ-FT, ThermoElectron, Corp., Waltham, MA). Five percent of each sample was injected onto a nano-LC column (75 μm ID × 10 cm, Atlantis dC18 RP, 3μm particle size, Waters Corp.) using a nano-LC system (NanoLC, Eksigent Technologies, Dublin, CA) with a gradient of 9% to 60% acetonitrile in 0.1% formic acid at 400 nL/min (Additional file 1: Table S1). Primary mass spectra were acquired in the FTMS (FT-ICR) portion of the instrument and MS/MS sequence information was collected in the linear ion trap using collision-induced dissociation (CID) to fragment peptides. Primary mass spectra were acquired with typically better than 2 ppm mass error; CID spectra were typically acquired with less than 0.3 Da mass error.

### Data analysis

Peaklists (i.e. DTA files) for database searching were generated for peptide precursor ions (i.e. +1, +2, and/or +3 charge states) and corresponding CID fragmentation data using SEQUEST (BioWorks Browser, revision 3.2, ThermoElectron Corp.) with the default parameters. Resulting DTA files from each sample acquisition were combined for each study group and analyzed using MASCOT (software version 2.1.03, Matrix Science, Inc., Boston, MA). The dataset of malto-oligosaccharide-solubilized glycogen-associated proteins had 26,102 queries, with 11,070 queries occurring in the control maltodextrin-insoluble glycogen pellet preparation. Both datasets were searched using the “mammalia” taxonomy classification within the MSDB database (down loaded 08/06/2007, with 3239079 sequences, 339491 after restricting to the “mammalia” taxonomy). The “mammalia” taxonomy was employed in order to detect contamination from human keratins and porcine trypsin. It was also sufficiently large to provide a good estimate of the false discovery rate (FDR). The following MASCOT search parameters were used in the analysis: tryptic-specific peptides, maximum of 3 missed cleavages, mass tolerances of 5 ppm for precursor ions and 0.3 Da for MS/MS CID fragment ions, no fixed modifications, and variable oxidation (M), phosphorylation (STY) and O-linked N-acetylglucosamine (ST). No phosphorylated or glycosylated peptide were identified in the final dataset. A significance threshold of p < 0.05 for identified proteins was used. All individually identified peptides with expectation scores above 0.05 were excluded. Both parameters are appropriate as being equivalent to statistical significance [67]. To gain a conservative estimate of the false discovery rate (FDR) a decoy database was developed by the MASCOT software. The FDR is calculated as the number of false positives (FP), determined by searching the decoy database, divided by the number of matches (M) in the target “mammalia” database (FDR = FP/M). The decoy database consisted of random sequences, of the same length and average amino acid distribution of the “mammalia” database, generated for each identified peptide. The same criteria for acceptance was employed for both target and decoy databases. Searches of the mammalian database resulted in identification of 2340 peptide sequences from the datasets, which satisfied the Mascot “identity threshold”. Searches of the “decoy” database using the same parameters based on these datasets resulted in 91 matches, giving an FDR of 3.8%. Employing the same protocol on the control maltodextrin-insoluble dataset resulted in 1114 and 77 peptide assignments from the forward and “decoy” databases respectively, with an FDR of 6.9%. For the present study, in order for a protein identification to be considered valid, at least two unique peptides, with different primary sequences and expectation scores less than 0.05, were required to be identified from a gene product in the protein reference database.

In order to achieve consistent protein assignment, the Mascot search results were exported in the comma-delimited CSV format and arrayed in a spreadsheet (Microsoft ^®^ Excel ^®^ 2004 for Mac Version 11.3.7). Peptides from poorly annotated protein assignments were submitted to the International Protein Index (IPI) mouse or rat database (April 2008) using the PROWL website (http://prowl.rockefeller.edu). The Swiss-Prot identifier was obtained and submitted to the UNIPROT database (http://www.uniprot.org) since it is the most annotated. The resulting UNIPROT entries were used to assign identifiers and obtain sub-cellular distribution data for each identified gene product.

## Abbreviations

ACN: Acetonitrile; AMPK: AMP-activated protein kinase; CBM20: Carbohydrate binding module_family 20; CBM48: Carbohydrate binding module_family 48; CID: Collision-induced dissociation; DTA: SEQUEST peaklist data file format; emPAI: Exponentially modified Protein Abundance Index; FDR: False Discovery Rate; FP: False Positive; FTMS: Fourier transform mass spectrometry; G6P: Glucose-6-phosphate; MO: Malto-oligosaccharide; MS: Mass spectrometry.

## Competing interests

The authors declare no competing interests in this study.

## Authors’ contributions

DS, DM and GP conceived the studies; DS and GP designed the experiments; DS, MF and GP wrote the manuscript. GP isolated the glycogen-associated proteins from 3T3-L1 cells; CN and KP identified the proteins by mass spectrometry; CN, KP and GP performed the data analyses. All authors read and approved the final manuscript.

## Supplementary Material

Additional file 1: Table S1 The Adipocyte Glycogen Proteome. Click here for file
