# Supplementary material for: The 3T3-L1 adipocyte glycogen proteome
Source: Proteome Sci. 2013 Mar 22;11:11. doi: 10.1186/1477-5956-11-11 (PMC3622581; doi:10.1186/1477-5956-11-11)
Supplement: Additional file 1: Table S1 — The Adipocyte Glycogen Proteome. [file 1477-5956-11-11-S1.doc]

SUPPLEMENTAL DATA

**Supplemental Table 1. The Adipocyte Glycogen Proteome**

Proteins isolated from glycogen particles of mouse 3T3-L1 adipocytes were solubilized by malto-oligosaccharide treatment and trypsinized. Peptides were desalted, purified, and analyzed by mass spectroscopy (LC/FTMS/MS) and bioinformatic searches of the “mammalia” MSDB database. Mouse proteins were identified by name, UNIPROT identifier (ID), and primary citable accession number. Proteins that that meet a significance level of less than 0.05 and with at least two different unique peptide sequences with expectation scores less than 0.05, are listed in order of likelihood by Mowse score (score) (1). Other parameters include: the total number of peptides with expectation scores less than 0.05 (total), number of different unique peptide sequences identified corresponding to the specific gene product (#), percent coverage of the protein (%), the lowest recorded expectation value (E-value) for a unique non-redundant peptide from the identified protein, the exponentially-modified Protein Abundance Index value (emPAI), and cellular compartment as annotated in the UNIPROT data base ([www.uniprot.org](http://www.uniprot.org/)) (2). In the case of proteins with homology to other glycogen-associated proteins, the number of unique, non-redundant peptides is indicated in parentheses. *The UniProt listing for GDE-MOUSE is not complete therefore the parameters presented correspond to the closet match in the database, GDE_RABIT. †Insufficient data was obtained to conclusively match the identified peptides with a unique gene product.

Supplemental Table 1.

References

1. Pappin, D. J., Hojrup, P., and Bleasby, A. J. (1993) *Curr Biol* **3**(6), 327-332

2. Ishihama, Y., Oda, Y., Tabata, T., Sato, T., Nagasu, T., Rappsilber, J., and Mann, M. (2005) *Mol Cell Proteomics* **4**(9), 1265-1272
